# The association between nonalcoholic fatty liver disease and corrected QT interval prolongation among generally healthy Iranian population: Fasa Cohort Study (FACS)

**DOI:** 10.1002/clc.24015

**Published:** 2023-04-04

**Authors:** Alireza Naderi, Mojtaba Farjam, Maryam Mojarrad Sani, Ashkan Abdollahi, Abdulhakim Alkamel, Omid Keshavarzian, Reza Tabrizi

**Affiliations:** ^1^ Department of Medicine, USERN Office Fasa University of Medical Sciences Fasa Iran; ^2^ Noncommunicable Diseases Research Center Fasa University of Medical Science Fasa Iran; ^3^ Department of Medicine Tehran University of Medical Sciences Tehran Iran; ^4^ Department of Medicine Shiraz University of Medical Sciences Shiraz Iran; ^5^ Clinical Research Development Unit Valiasr Hospital, Fasa University of Medical Sciences Fasa Iran

**Keywords:** cardiovascular mortality, general healthy Iranian population, nonalcoholic fatty liver disease, QTc interval prolongation

## Abstract

**Background:**

There are limited studies about the association between nonalcoholic fatty liver disease (NAFLD) and corrected QT interval (QTc) prolongation worldwide.

**Hypothesis:**

Therefore, we designed the current study to determine this association in a large cohort of a generally healthy population.

**Methods:**

We analyzed the data of 4603 individuals aged 35–70 who participated in the Fasa Cohort Study (FACS). Based on 12‐lead electrocardiograms, QT intervals were calculated and corrected by Bazzet's formula. A QTc interval of more than 430 ms in men and 450 ms in women was considered prolonged. The Fatty Liver Index was used to identify the participants with NAFLD.

**Results:**

Of all participants, 1550 (33.6%) met the NAFLD criteria. In subjects of both genders with NAFLD, the mean values of the QTc interval were considerably higher than in those without NAFLD (*p* < .001). After adjusting for a wide range of confounders, including age, gender, smoking status, physical activity, total cholesterol, high‐density lipoprotein‐cholesterol levels, diabetes, and hypertension status, in linear regression analysis, the standardized β coefficient of QTc interval among participants with NAFLD was 2.56 ms (95% confidence interval [CI]: 0.49–4.64). After controlling the same confounders, the odds ratio of NAFLD for a prolonged QTc interval in men was 1.47 (95% CI: 1.18–1.84; *p* < .001) and in women was 1.39 (95% CI: 1.15–1.68; *p* < .001) using logistic regression analysis.

**Conclusions:**

NAFLD was a risk factor for QTc interval prolongation. Awareness about the risk of NAFLD in increasing the potential cardiac arrhythmias should be raised to lower cardiac mortality.

## Graphical abstract

We studied the data of 4603 individuals. In subjects with NAFLD, the QTc interval is, on average, 2.56 ms longer than in those without NAFLD. The chance of QTc interval prolongation among males and females with NAFLD is 1.47 and 1.39 folds more than those without NAFLD, respectively. As QTc prolongation is associated with cardiac mortality, monitoring individuals with NAFLD is crucial.

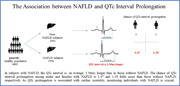

**Abbreviations**
ALT: alanine transaminase
AST: aspartate aminotransferase
BMI: body mass index
CI: confidence interval
FACS: Fasa Cohort Study
FBS: fasting blood sugar
GGT: gamma‐glutamyl transferase
HDL: high‐density lipoprotein
IPAQ: International Physical Activity Questionnaire
LDL: low‐density lipoprotein
NAFLD: nonalcoholic fatty liver disease
TG: triglyceride

## 1 INTRODUCTION

Nonalcoholic fatty liver disease (NAFLD) was initially described as “a less known and until now an anonymous hepatic disease.”^1^ Our understanding of this condition has quickly grown during the last few decades. Without excessive alcohol consumption or viral hepatitis, NAFLD is now defined as the accumulation of macrovesicular steatosis in more than 5% of hepatic cells.^2^ This disease includes a spectrum ranging from steatosis to nonalcoholic steatohepatitis, which can lead to liver fibrosis and cirrhosis.^3^ NAFLD is the most common cause of chronic liver dysfunction. NAFLD affects 25.24% of the population overall and has links to metabolic risk factors such as diabetes, hypertension, dyslipidemia, and obesity, according to a meta‐analysis study including 22 countries.^4^, ^5^ The prevalence of NAFLD has gradually increased in Asia due to lifestyle changes.^6^ Furthermore, NAFLD is an emerging health problem in Iran, with a total prevalence of 33.9%.^7^

The time between ventricular depolarization and repolarization in the ECG is known as the QT interval. It is measured as the time from the start of the QRS complex to the end of the T wave. Due to the effect of heart rate (HR) on QT interval, a corrected QT interval (QTc) adjusted for the HR is mainly used.^8^ Prolonged QTc interval in ECG is a well‐known risk factor for ventricular arrhythmia, tachycardia, and sudden cardiac death (SCD) in people with cardiovascular risk factors and otherwise healthy populations.^9^, ^10^, ^11^ Notably, the duration of the QT interval, even within a range of reference, predicts cardiovascular death in the general population.^10^, ^12^

The association between NAFLD and an increased risk of cardiovascular mortality has been reported in previous studies.^13^ The QTc interval prolongation, which can result in ventricular arrhythmia and SCD, could partly explain this increased risk in patients with NAFLD.^9^, ^10^ Even though previous studies have reported the association between NAFLD and prolonged QTc interval, most have been conducted in Western or far East specific populations such as those with chronic medical conditions, including type 2 diabetes mellitus, women, or steelworkers. For diagnosis of NAFLD, all similar studies used abdominal ultrasonography, which is operator dependent, qualitative, and may be less accessible than Fatty Liver Index (FLI) in health centers.^14^, ^15^ Moreover, factors such as underlying diseases, ethnicity, lifestyle, and diet might influence the results, and FLI is a quantitative, easy‐to‐employ^14^ parameter for diagnosing NAFLD. Therefore, using the FLI score, we evaluated the association between NAFLD and prolonged QTc interval among the generally healthy Iranian population.

## 2 METHODS

### 2.1 Study population

**Figure 1:**
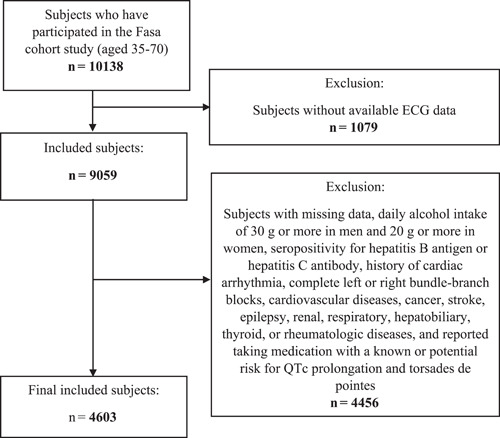
Study enrollment flowchart.

The Fasa Cohort Study (FACS) enrolled 10 138 patients aged between 35 and 70 from October 2014 to September 2016. Details of the study's design and methods are described elsewhere.^16^ People were invited by healthcare experts in the rural health system of Sheshdeh district and its 24 surrounding villages to participate in this study.^16^ At the start of the study, all participants provided written informed consent. Our sample included all subjects with available ECG data (*n* = 9059). Subjects who met one or more of the following criteria were excluded (*n* = 4456): missing data, alcohol intake of 30 g/day or more in men and 20 g/day or more in women (safety levels of consumption to prevent Alcoholic Liver Disease),^17^ having underlying diseases and reported taking medication with a known or potential risk for QTc prolongation.^18^ Finally, the analysis included a total of 4603 subjects. Figure 1 summarizes the study enrollment flowchart with more details.

### 2.2 Data acquisition

Each participant completed an internet‐based questionnaire that collected basic information such as age, gender, smoking status, history of alcohol consumption, and chronic diseases such as diabetes and hypertension.

All medication taken within the past 2 weeks before registration was asked about and recorded. According to CredibleMeds, a database of drugs with a risk of QT prolongation, drugs with a known or potential risk for torsades de pointes have been listed and used to identify the taken medication with influential QTc prolongation factors.^18^ A 20‐item questionnaire based on the International Physical Activity Questionnaire (IPAQ)^16^, ^19^ was used to assess rural Iranians' routine physical activity.

The MET‐value of each activity was multiplied by its duration to calculate MET‐min. Total physical activity (MET/24 h) was the sum of all activities. The average daily intake of 125 food parameters was measured per a validated quantitative Food Frequency Questionnaire (FFQ)^16^, ^20^ for energy intake assessment, and the results were reported in kcal/day.

### 2.3 Measurements

For anthropometric calculations, height was measured by a stadiometer, and a digital scale measured weight with an accuracy of 0.1 cm and 0.1 kg, respectively. Body mass index (BMI) was calculated as weight in kilograms divided by the square of the body height in meters (kg/m^2^). Waist circumference (WC) was measured by a tape measure at the midpoint of the inferior border of the lowest rib to the anterior superior iliac spine.

After a 15‐min rest, participants' blood pressure was measured while seated, using the individuals' right arm consecutively two times with an interval of 5 min. Then the average systolic and diastolic blood pressure was reported in mmHg.

### 2.4 Blood chemistry

After fasting for 10–14 h, all participants had the following blood chemistry tests: fasting blood sugar (FBS), triglyceride (TG), high‐density lipoprotein (HDL) cholesterol, low‐density lipoprotein (LDL) cholesterol, gamma‐glutamyl transferase (GTT), total cholesterol, aspartate aminotransferase (AST), and alanine transferase (ALT). The Mindray BS380 autoanalyzer measured total cholesterol and HDL (Mindray Medical International). TG was determined by enzymatic method and reported in mg/dL. AST and ALT were measured by the kinetic method and GGT by an enzymatic method based on SZASZ's approach. A glucose oxidase test was used for laboratory testing of FBS. Moreover, the Friedewald formula was used for the assessment of LDL.

### 2.5 Electrocardiogram

An expert technician obtained a 12‐lead ECG from each participant after they rested for 15 min. A computer‐based device (Cardiax®) performed ECGs. Participants were asked to shave the precordium area and refrain from moving or talking for better electrode attachment. The computer software (Cardiax®, version 3.50.2; International Medical Equipment Developing Co. Ltd.) interpreted and reported the ECGs, then exported them to the central data collection software. We used ECG data that includes HR (bpm), QT interval duration (ms), and QTc interval duration (ms), which were approved by a cardiologist.^16^ The QT interval (QTc) was calculated using Bazett's formula (QTc = QT/(RR)^1/2^).^21^ Cut‐off values for prolonged QTc were considered >430 ms for men and >450 ms for women.^10^

### 2.6 Definition of NAFLD

FLI, a cost‐effective, noninvasive method, was used to determine participants with or without NAFLD as a standard predictor of hepatic steatosis severity. This score is calculated using BMI, serum TG levels, WC, and GGT by the following formula^14^:

(1) $$\mathrm{FLI}=(e^{0.953}\times \log_{e}\;(\mathrm{TG})\;+0.139\times \mathrm{BMI}+0.718\times \log_{e}\;(\mathrm{GGT})+0.053\times \mathrm{WC}-15.745)/(1+e^{0.953}\times \log_{e}\;\;(\mathrm{TG})+0.139\times \mathrm{BMI}+0.718\times \log_{e}\;(\mathrm{GGT})+0.053\times \mathrm{WC}-15.745)\;\times 100.$$

Optimal cut‐off values of FLI in Iranian society have been assumed to be 46.9 in men (sensitivity 0.824, specificity 0.768) and 53.8 in women (sensitivity 0.823, specificity 0.765).^22^ Therefore, men with a score of more than 46.9 and women with a score of more than 53.8 were assumed to have NAFLD.

### 2.7 Statistical analysis

Data were presented as mean (SD) or median (interquartile range [IQR]) for continuous variables based on normality and counts and percentages for categorical variables. Comparisons were made using the independent Student's *t* test or Mann–Whitney *U* test, as appropriate, for continuous variables and *χ* ^2^ for categorical variables. Linear regression analysis determined the relationship between NAFLD and QTc interval duration. Logistic regression analysis was performed to determine the association between NAFLD and normal or prolonged QTc. Standardized β coefficient (β) in linear regression analysis was calculated. Logistic regression analysis calculated the odds ratio (OR) and 95% confidence interval (95% CI). Then, different regression models were adjusted using the most critical confounders, including age, sex, smoking status, physical activity, total cholesterol, HDL‐C, AST, ALT, diabetes mellitus, and hypertension history. *p* Values less than .05 were considered statistically significant. All statistical analyses were performed using SPSS 26.0 (IBM Corp.).

### 2.8 Ethics approval and consent to participate

Our study protocol adhered to the Helsinki Declaration, and the Fasa University of Medical Sciences Research Council and Ethics Committee approved it (approval code: IR.FUMS.REC.1400.117). In addition, the subjects provided written informed consent to participate in this study.

## 3 RESULTS

Four thousandsix hundred and three participants were studied, with 2095 (45.5%) males and 2508 (54.5%) females. The mean age was 47.65 years old (SD 9.10 years). Table 1 shows the clinical characteristics of the study population based on the presence of NAFLD. One thousand five hundred and fifty participants (33.6%) met NAFLD criteria based on the FLI. Systolic blood pressure, diastolic blood pressure, WC, BMI, FBS, TG, LDL, GGT, total cholesterol, AST, and ALT levels were significantly higher in NAFLD participants than in non‐NAFLD participants (*p* < .001). Women comprised more than half of the NAFLD participants (56.9%). The prevalence of diabetes and hypertension was higher, while smoking was lower in NAFLD participants than non‐NAFLD participants (*p* < .001). The amount of physical activity was significantly lower in subjects who had NAFLD (*p* < .001). As shown in Table 1, there is no statistically significant difference between participants with and without NAFLD in terms of age, energy intake (kcal/day), and history of alcohol consumption less than 30 g/day in men and 20 g/day in women (*p* > .05).

**Table 1:** Clinical characteristics of the study population based on the presence of NAFLD.

Variables	Total (4603)	NAFLD (+) (1550)	NAFLD (−) (3053)	*p* Value
Age (year)	47.65 ± 9.10	47.55 ± 8.62	46.70 ± 9.28	.572
Sex (male)	2095 (45.5%)	668 (43.1%)	1427 (46.7%)	.019
Systolic blood pressure (mmHg)	109.84 ± 17.23	115.03 ± 17.04	107.21 ± 16.73	<.001
Diastolic blood pressure (mmHg)	73.79 ± 11.30	77.55 ± 11.26	71.89 ± 10.83	<.001
Diabetes history	469 (10.2%)	234 (15.1%)	235 (7.7%)	<.001
Hypertension history	639 (13.9%)	317 (20.5%)	322 (10.5%)	<.001
Waist circumference (cm)	92.81 ± 11.62	103.83 ± 8.56	87.22 ± 8.57	<.001
BMI (kg/m^2^)	25.58 ± 4.81	30.20 ± 3.74	23.24 ± 3.40	<.001
Physical activity (Met/24 h)	41.77 ± 11.03	39.62 ± 9.47	42.85 ± 11.59	<.001
Active smoker	1071 (23.3%)	271 (17.5%)	800 (26.2%)	<.001
Alcohol consumption history	68 (1.5%)	15 (1%)	53 (1.7%)	.520
Energy intake (kcal/day)	2942.51 ± 1116.52	2972.0 ± 1141.37	2927.54 ± 1103.58	.202
FBS (mg/dL)	92.17 ± 29.17	97.20 ± 3474	89.61 ± 25.52	<.001
TG (mg/dL)	131.78 ± 83.98	185.12 ± 110.67	104.71 ± 47.31	<.001
HDL (mg/dL)	48.26 ± 12.83	45.69 ± 12.43	49.56 ± 12.84	<.001
LDL (mg/dL)	110.85 ± 31.64	118.75 ± 33.33	106.83 ± 29.97	<.001
GGT (U/L)	21.95 ± 19.98	29.78 ± 20.94	17.97 ± 18.23	<.001
Total cholesterol (mg/dL)	185.50 ± 38.72	201.52 ± 38.85	177.36 ± 36.2	<.001
AST	22.31 ± 8.30	23.67 ± 9.28	21.62 ± 7.66	<.001
ALT	23.17 ± 14.01	28.86 ± 16.87	20.27 ± 11.25	<.001

*Note*: Data are presented as mean ± standard deviation or number (%). *p* Values are based on independent samples *t* test and *χ* ^2^.

Abbreviations: ALT, alanine transaminase; AST, aspartate aminotransferase; BMI, body mass index; FBS, fasting blood sugar; GGT, gamma‐glutamyl transferase; HDL, high‐density lipoprotein; LDL, low‐density lipoprotein; NAFLD, nonalcoholic fatty liver disease; TG, triglyceride.

Table 2 shows the ECG parameters of the study population based on the presence of NAFLD in both genders. According to this table, males with NAFLD have significantly higher HR and QTc interval values than males without NAFLD (*p* < .001 for both). On the contrary, the QT interval in males with NAFLD was significantly lower than in males without (*p* < .001). Among females, HR and QTc interval were significantly higher in participants with NAFLD than those without NAFLD (*p* < .001, and *p* = .017, respectively). Furthermore, although the QT interval in females with NAFLD was shorter than in those without NAFLD, the difference was not statistically significant (*p* = .154).

**Table 2:** ECG parameters of the study population according to the presence of NAFLD in both genders.

	Male (*n* = 2095)			Female (*n* = 2508)		
	NAFLD (+)	NAFLD (−)	*p* Value	NAFLD (+)	NAFLD (−)	*p* Value
HR (bpm)	69.37 (10.77)	64.47 (10.38)	<.001	76.58 (11.52)	74.60 (11.17)	<.001
QT (ms)	395.93 (31.67)	405.18 (34.42)	<.001	392.46 (36.17)	394.63 (36.43)	.154
QTc (ms)	422.86 (28.69)	416.97 (29.80)	<.001	440.39 (32.63)	437.08 (33.29)	.017

*Note*: Data are presented as mean ± standard deviation or number (%). *p* Values are based on independent samples *t* test and *χ* ^2^.

Abbreviations: HR, heart rate; NAFLD, nonalcoholic fatty liver disease; QTc, corrected QT interval.

Table 3 summarizes the results of multiple linear regression analyses of the independent association between NAFLD and QTc interval in the general population. Table 4 shows the results of multiple logistic analyses of the independent association between NAFLD and QTc interval in males and females separately. After adjusting for age, gender, smoking status, physical activity (MET 24 h), total cholesterol, HDL‐cholesterol, AST, ALT levels, diabetes, and hypertension status, our multiple linear regression analysis shows a significantly positive association between the presence of NAFLD and an increase in QTc increment in both genders (standardized β coefficient = 2.56 ms, 95% CI: 0.49–4.64; *p* = .015). According to the multiple logistic regression analysis, there is a significantly positive relationship between the presence of NAFLD and a prolonged QTc interval in males >430 ms (OR = 1.47, 95% CI: 1.18–1.84; *p* < .001) and females >450 ms (OR = 1.39, 95% CI: 1.15–1.68; *p* < .001) after adjusting for age, smoking status, physical activity (MET 24 h), total cholesterol, HDL‐cholesterol, ASL, ALT levels, and diabetes and hypertension status.

**Table 3:** Multiple linear regression analysis findings to assess the association of NAFLD with QTc and prolonged QTc interval.

QTc (ms)		Multiple linear regression		
		*β*	95% CI	*p*
Unadjusted		5.15	3.14, 7.16	<.001
Adjusted				
Model 1		4.49	2.58, 6.41	<.001
Model 2		3.73	1.80, 5.65	<.001
Model 3		2.56	0.49, 4.64	.015

*Note*: Data were analyzed using Multiple linear regression analysis with Forward methods. Model 1: adjusted for age and sex. Model 2: adjusted for age, sex, smoking status, and physical activity (MET 24 h). Model 3: adjusted for age, sex, smoking status, physical activity (MET 24 h), total cholesterol, HDL‐cholesterol, AST, ALT levels, diabetes, and hypertension.

Abbreviations: ALT, alanine transaminase; AST, aspartate aminotransferase; CI, confidence interval; HDL, high‐density lipoprotein; NAFLD, nonalcoholic fatty liver disease; QTc, corrected QT interval.

**Table 4:** Multiple logistic regression analysis findings to assess the association of NAFLD with QTc and prolonged QTc interval.

			Multiple logistic regression		
			Odds ratio	95% CI	*p*
*Prolonged QTc in males (>430 ms)*					
Unadjusted			1.58	1.29, 1.92	<.001
Adjusted					
Model 1			1.66	1.35, 2.03	<.001
Model 2			1.61	1.31, 1.98	<.001
Model 3			1.47	1.18, 1.84	<.001
*Prolonged QTc in females (>450 ms)*					
Unadjusted			1.44	1.20, 1.74	<.001
Adjusted					
Model 1			1.44	1.20, 1.73	<.001
Model 2			1.42	1.18, 1.71	<.001
Model 3			1.39	1.15, 1.68	<.001

*Note*: Data were analyzed using Multiple logistic regression analyses with Forward methods. Model 1: adjusted for age. Model 2: adjusted for age, smoking status, and physical activity (MET 24 h). Model 3: adjusted for age, smoking status, physical activity (MET 24 h), total cholesterol, HDL‐cholesterol, AST, ALT levels, diabetes, and hypertension.

Abbreviations: ALT, alanine transaminase; AST, aspartate aminotransferase; CI, confidence interval; HDL, high‐density lipoprotein; NAFLD, nonalcoholic fatty liver disease; QTc, corrected QT interval.

## 4 DISCUSSION

We investigated the association between NAFLD and prolonged QTc interval in a large cohort of a generally healthy population in southern Iran. After controlling for a wide range of potentially confounding variables, our study found a positive relationship between NAFLD and prolonged QTc interval in the aimed population.

Our findings are consistent with the previous studies investigating the association between NAFLD and QTc prolongation.^23^, ^24^, ^25^, ^26^ According to a study by Targhar et al.^23^ conducted on 400 Italian participants, the presence and severity of NAFLD had an association with increased QTc interval in type 2 DM patients with an adjusted OR of 2.26 (95% CI: 1.4–3.7). In another study consisting of 31 116 Taiwanese cases,^25^ NAFLD severity had a positive correlation with prolonged QTc interval in both genders [adjusted OR in men 1.87 (1.51–2.31) and in women 1.31 (1.16–2.24)]. A study of 764 apparently healthy Korean women reported the same results with an OR of 2.05 (1.13–3.71).^26^ Furthermore, in a study of 2998 Chinese male steelworkers, Hung et al.^24^ reported a positive association of NAFLD severity with QTc prolongation [adjusted OR: 2.54 (1.22–5.39)].

Although previous studies in Western or far East Asian countries with mostly fewer subjects found a significantly positive association between NAFLD and an increased risk of QTc prolongation, this is the first study in Western Asia. In this study, we confirmed the positive relationship between NAFLD and a prolonged QTc interval of 4603 generally healthy Iranians. There are several possible explanations for the association between NAFLD and QTc prolongation. Diabetes, smoking, systolic and diastolic blood pressure, hemoglobin A1c, high‐density lipoprotein concentration, BMI, and WBC count are associated with developing QTc prolongation in individuals with NAFLD.^10^, ^24^, ^25^ Inflammatory processes could be another underlying mechanism. NAFLD is caused by a low‐grade inflammatory response mediated by different cytokines, which can affect the myocardium and cause an increase in action potential duration and QTc interval.^27^ TNF‐α has been implicated as a proinflammatory cytokine in the induction of calcium leakage from the sarcoplasmic reticulum, which leads to QTc prolongation and arrhythmia.^28^ The third mechanism to explain the association between NAFLD and QTc prolongation may be the presence of sympathetic hyperactivity in NAFLD participants. The reason is that the sympathetic nervous system is associated with QTc prolongation,^29^ and people with NAFLD are more likely to experience autonomic imbalance.^29^, ^30^ However, further studies are needed to elucidate the exact relationship mechanism between NAFLD and QTc prolongation.

Our findings could improve clinical management and outcomes in patients with NAFLD or cardiovascular diseases. Nevertheless, there are also some limitations to our study. First, because this was an observational study, we needed to determine a possible causal relationship between NAFLD and QTc prolongation. Second, although FLI has good sensitivity and specificity for detecting NAFLD compared with abdominal ultrasonography, it has a lower sensitivity and specificity than liver biopsy, which is the gold standard for NAFLD diagnosis.^22^, ^31^ As a result, the prevalence of NAFLD in our study may be underestimated, which is ignorable. However, the large sample size is a significant strength of the current study. We were able to assess the association between NAFLD and QTc prolongation more accurately, owing to the extensive measures of confounding factors in the FACS and the highly standardized procedures for obtaining digital ECG data. Furthermore, this kind of investigation in Western Asia is novel to our knowledge.

## 5 CONCLUSION

We confirmed the relationship between NAFLD and QTc interval prolongation in a generally healthy Iranian population. As QTc prolongation is associated with an increased risk of ventricular arrhythmia, and SCD, we recommend that individuals with NAFLD change their lifestyle and undergo regular ECG monitoring. Early identification of QTc prolongation in the electrocardiogram, as a predictor of potential cardiac arrhythmias, can lead to early treatment, consequently lowering the risk of cardiac mortality.

## CONFLICT OF INTEREST STATEMENT

The authors declare no conflict of interest.

## Data Availability

The authors confirm that the data supporting the findings of this study are available within the article. Raw data supporting this study's results are available from the corresponding author upon reasonable request.
